# Assessing microbial manipulation and environmental pollutants in the pathogenesis of psoriasis

**DOI:** 10.3389/fimmu.2022.1094376

**Published:** 2022-12-20

**Authors:** Portia Gough, Jordan Zeldin, Ian A. Myles

**Affiliations:** Epithelial Therapeutics Unit, National Institute of Allergy and Infectious Disease, National Institutes of Health, Bethesda, MD, United States

**Keywords:** psoriasis, microbiome, *Roseomonas*, atopic dermatatis, pollution

## Abstract

The cutaneous microbiome is increasingly recognized as a contributor to skin diseases like atopic dermatitis (AD) and psoriasis. Although traditionally AD and psoriasis have been viewed as having opposing immunologic findings, recent evidence suggests an overlap in ceramide-family lipid production in the protection against symptoms. We recently identified that specific environmental pollutants may drive dysbiosis through direct suppression of ceramide-family lipids produced by health-associated skin bacteria in atopic dermatitis (AD). We further demonstrated that one such bacteria, *Roseomonas mucosa*, generated significant clinical improvement in AD lasting beyond active treatment *via* lipid-mediated modulation of tumor necrosis factor (TNF) signaling. To assess the potential preclinical benefit of *R. mucosa* in psoriasis we assessed for direct effects on surface TNF signaling in cell cultures and identified direct effects on the TNF axis. We also identified preclinical efficacy of *R. mucosa* treatment in the imiquimod mouse model of psoriasis. Finally, we expanded our previous environmental assessment for psoriasis to include more traditional markers of air quality and found a strong association between disease rates and ambient carbon monoxide (CO), nitrogen dioxide (NO_2_), and particulate matter (PM). At the current stage this work is speculative but does support consideration of further preclinical models and/or clinical assessments to evaluate any potential for therapeutic benefit through microbial manipulation and/or environmental mitigation.

## 1 Introduction

The cutaneous microbiome is increasingly recognized as a contributor to skin disease. Although the literature is less robust for the skin microbiome in psoriasis as it is for related gut dysbiosis or dysbiosis in diseases like atopic dermatitis (AD), both microbial signatures and therapeutic targeting have been described (1–3). We recently suggested that environmental pollutants may drive dysbiosis through direct effects on cutaneous commensal bacteria (4). Furthermore, we have reported that use of topical *Roseomonas mucosa* generated significant clinical improvement in AD lasting beyond active treatment (4, 5). These lasting effects were likely related to colonization with the treatment strains whose modeled mechanism involves the production of amine-containing lipids (such as ceramides) which potentiated tumor necrosis factor (TNF) receptor 2 (TNFR2) signaling to drive epithelial repair (5, 6). For AD, these lipids appeared to be directly depleted by exposure to the isocyanate containing air and may suggest a mechanism for the association between industrial environments and AD-linked dysbiosis (4).

However, unlike psoriasis (7, 8), TNF has not classically been considered a central mediator in AD. Yet, psoriasis and AD have both been linked to a reduction in cutaneous ceramide levels (9). Furthermore, epidemiologic features that argue for a potential environmental contributor to psoriasis include: finding that the rates in the US have increased 1.75 fold since 1974 (10); urban living carries an odds ratio of 3.61 compared to rural (11); tobacco, alcohol, and change in work or family conditions increase risk of psoriasis (11); and rates are higher in high income countries (although data in many nations are lacking) (12). Although our preliminary screen of pollutants that may contribute to psoriasis failed to identify any strong candidates (4), we hypothesized that *R. mucosa* treatment could benefit psoriasis through impacts on TNFR2 signaling and/or production of therapeutic lipids.

Due to the shared pathology of TNF and ceramide family lipids, we thus aimed to assess if R. mucosa directly influences the TNF axis *in vitro* and models of psoriasis *in vivo*. Furthermore, given the associations between industrialization and psoriasis, we aimed to perform an untargeted assessment of Environmental Protection Agency (EPA) monitored pollutants versus the rate of clinical visits for psoriasis by US zip codes. Herein we describe that *R. mucosa* directly influences surface TNF, TNFR2, and TNF alpha converting enzyme (TACE, aka ADAM17) in cell culture models and influenced soluble TNFR2 in patients with AD. *R. mucosa* improved outcomes in the imiquimod (IMQ) model of murine psoriasis, a model known to be dependent on TNFR signaling balance (7). Finally, we expanded our previous environmental assessment for psoriasis to include more traditional markers of air quality and found a strong association between disease rates and ambient carbon monoxide (CO), nitrogen dioxide (NO2), and particulate matter (PM). Overall, our findings support the consideration of clinical trials using *R. mucosa* for psoriasis as well as further *in vitro* modeling for a role of air pollution in psoriasis pathogenesis.

## 2 Methods

### 2.1 Cell culture and staining

Primary Neonatal keratinocytes (HEKn, ATCC) were seeded at 1.5x10^5^ cells/well in 24 well plates that were coated with 10 µg/mL human fibronectin (Millipore). The cells were allowed to settle for 1 hour. RmHV1 were added at MOI 10, and cells were incubated at 37°C and 5% CO2 for 12 hours. Cells were fixed in 4% PFA, blocked with 10% normal goat serum, and labeled with antibodies for TNFR2 (Sigma, SAB4502989, 1:100), TACE/ADAM17 (ThermoFisher, 3H46L1, 1:200), or TNF (Abcam, ab1793, 1:100) overnight at 4°C. Cells were washed and secondary antibodies (anti-rabbit AlexaFluor 488 or anti-mouse AlexaFluor 594, ThermoFisher, 1:1000) were added and incubated for 1 hour at room temperature. Cells were washed and DAPI was added as counterstain (ThermoFisher). Cells were imaged in 4 different areas of well using a Cytation5, and image analysis was performed by Gen5 software as follows: each cell was identified based on nuclear stain, and fluorescence intensity was measured for each cell in image and was divided by total number of cells to obtain the mean fluorescence intensity (MFI) value for each image.

### 2.2 Patients

Patients were recruited and enrolled at the NIH under the IRB approved clinical trial NCT03018275 (Beginning Assessment of Cutaneous Treatment Efficacy of *Roseomonas* in Atopic Dermatitis, Phase I/II; BACTERiAD I/II). Enrollment criteria, treatment formulation, and clinical scoring were all as previously described (5).

### 2.3 Serum analysis

Serum samples were analyzed for sTNFR1 and sTNFR2 using the BioPlex system (BioRad, Hercules, CA) per manufacture instructions and as previously described (5).

### 2.4 Mice

Male and female C57BL/6 mice aged 6-12 weeks were purchased from Jackson Labs (Bar Harbor, MA). All mice were age and sex matched within each experiment. Imiquimod modeling of psoriasis was performed as previously described (13). Briefly, IMQ was applied to each ear daily for 5 days, then either 10mcL of 10% sucrose or 10^5^ CFU of *R. mucosa* in 10mcL of 10% sucrose was applied daily for 3 days. Ear thickness was monitored, and ears collected for histology analysis at day 11. Ear mounting to slides and H&E staining was performed by Historserv (Germantown, MD). Images of slides were collected as previously described (5). A histologic comparison of the ear pinna between the diluent-treated, RnAD-treated, and RmHV-treated mice (i.e. 3 total) was made based solely on the submitted microscopic image from each animal. Animal work was approved by an IACUC and followed the guidelines and basic principles in the United States Public Health Service Policy on Humane Care and Use of Laboratory Animals, and the Guide for the Care and Use of Laboratory Animals by certified staff in an Association for Assessment and Accreditation of Laboratory Animal Care (AAALAC) International accredited facility.

### 2.5 Pollution analysis

As detailed previously (4), data on pollution was derived from the Environmental Protection Agency’s Risk-Screening Environmental Indicators (RSEI) system from 2015-2019 was contrasted against the Definitive Health database, which contains 1.2 billion diagnostic billing codes per year from 2019 across 20,000 zip codes in the United States, including Hawaii, Alaska, and U.S. territories for ICD-10 code L40.9. Additional data on air pollution in 2015 from Center for Air, Climate, and Energy Solutions (CACES) was used to incorporate CO, NO_2_, particulate matter of 2.5 microns or less (PM2.5), PM10, and SO_2_ (14). For each zip code tabulation area where ICD codes are aggregated, the concentration of pollution from the surrounding census tracts within a 50-mile radius were averaged. Weights were based on the population in the census tract and the proximity to the zip code centroid. Other covariates included the population density, bracketed age ranges [from American Community Survey), the Area Deprivation Index (from Neighborhood Atlas (15)], and the proportion of visits to specialists (i.e., rheumatologists, dermatologists). The spatially lagged y autocovariate was constructed with inverse distance weights. Poisson regression lasso was implemented with the glmnet package using spatial folds created with 10 k-means clusters. Random forest regression was implemented with the ranger package, with case weights equal to the total number of visits, mtry untuned to 1/3^rd^ the number of features, and 1000 trees.

## 3 Results

### 3.1 *R. mucosa* directly influences cell surface TNF in keratinocytes

**Figure 1:**
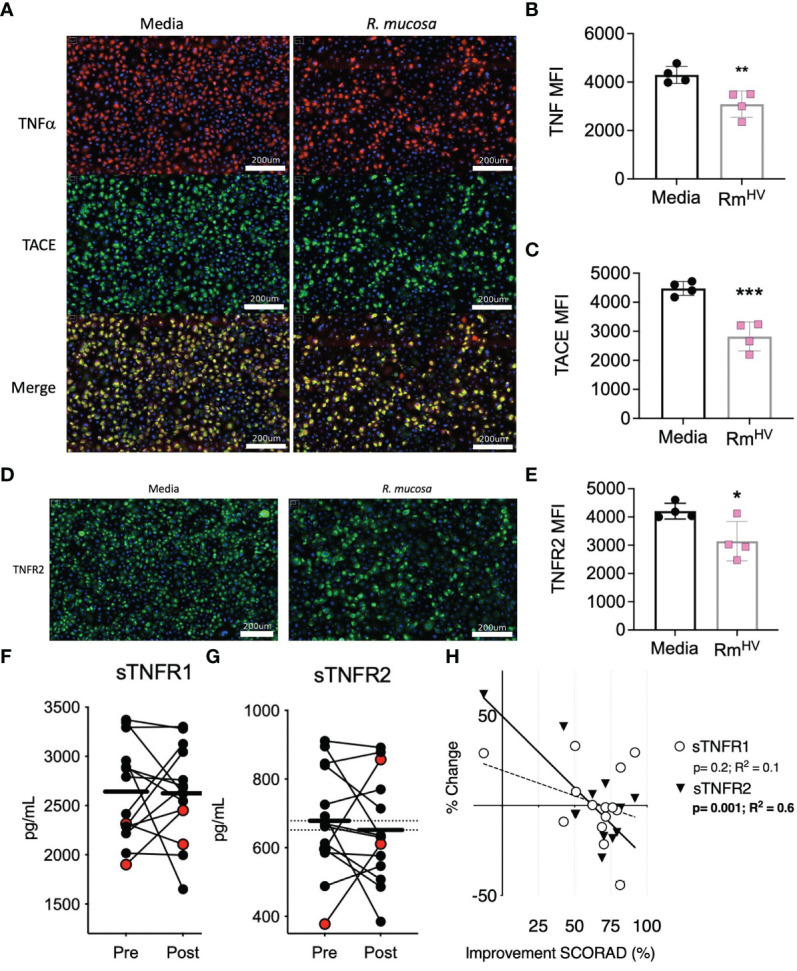
*R. mucosa* alters surface TNF-related marks in human keratinocyte cultures and patients with AD. **(A)** Representative images for immunofluorescent staining of tumor necrosis factor alpha (TNFα; red), TNFα converting enzyme (TACE; green) and both (merge; yellow) for human primary keratinocytes stimulated with *R. mucosa* from healthy volunteers or media alone. White lines represent scale of 200 micrometers. **(B, C)** Quantification of signal for indicated marker in replicate wells. **(D, E)** Representative image **(D)** and quantification in replicate wells **(E)** for immunofluorescent staining of tumor necrosis factor receptor 2 (TNFR2). White lines **(D)** represent scale of 200 micrometers. **(F–H)** 14 pediatric patients with atopic dermatitis (AD) were treated with topical *R. mucosa* for a total of four months. **(F, G)** Levels of soluble tumor necrosis factor receptor 1 (sTNFR1) and sTNFR2 in the serum at enrollment (Pre) or after 16 weeks of active treatment (Post) are shown. Red dots indicate the two patients that did not achieve at least a 50% improvement in symptoms during treatment. **(H)** Percent change in sTNFR1 and sTNFR2 levels are contrasted against improvement in SCORing AD are shown along with a simple linear regression line. Data represent three independent experiments and are displayed as mean ± SEM. ***p<0.001, **p<0.01, *p<0.05 as determined by Student T test.

We previously demonstrated the modeled activity of *R. mucosa* was dependent upon TNFR signaling, but such analysis could not discriminate if the bacteria directly impacted surface expression of TNF signaling or if the activity was intra-cellular. To assess this, we stained human primary keratinocytes for surface TNFα, TNFα converting enzyme (TACE), and TNFR2. After 12 hours of co-culture, the expression for each of these markers was significantly reduced by exposure to *R. mucosa*. (**Figures 1A–E**).

### 3.2 *R. mucosa* induced reduction of soluble TNFR2 associated with clinical improvement

We previously reported the impact of topical *R. mucosa* treatment on select serum cytokines in an open-label clinical protocol for patients with AD (5). In that initial report we identified reductions in serum TNF was associated with clinical improvement (5) but had not evaluated the balance of TNF receptor expression. Analysis of the samples revealed that treatment was associated with a significant reduction in soluble TNFR2 (sTNFR2) but not sTNFR1 (**Figures 1F, G**). Like TNF, reductions in sTNFR2 significantly associated with improvement in AD as measured by SCORing Atopic Dermatitis (SCORAD) metric (a metric which combines clinical scores for itch, rash, and sleep disturbance) (**Figure 1H**). Together, these data suggest that *R. mucosa* may have a direct impact on the cell surface TNF axis.

### 3.3 *R. mucosa* treatment improved outcomes in the IMQ murine model of psoriasis

**Figure 2:**
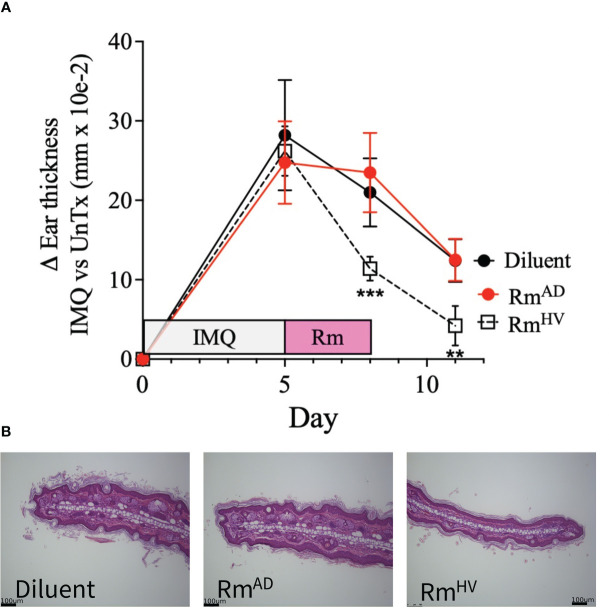
Topical *R. muocsa* improves mouse models of psoriasis. Mice (N = 5 per group) were treated with imiquimod (IMQ) daily for 5 days on one ears before topical treatment with 10^6^ colony forming units of R. mucosa from a healthy volunteer (Rm^HV^), a patients with atopic dermatitis (Rm^AD^), or diluent control were applied with the same ear for 3 days. **(A)** Differences between the unperturbed and treated ear are shown. **(B)** Representative images for one treated ear from each group are shown. Black bars indicate 100um scale. Data represent two independent experiments and displayed as mean ± SEM. ***p<0.001, **p<0.01.

Given that the IMQ model of psoriasis is dependent on TNFR signaling balance (7) and cutaneous ceramide levels (9) we hypothesized that *R. mucosa* treatment may improve outcomes. All groups presented with the expected histopathologic features of IMQ exposure (16) including epidermal hyperplasia and hyperkeratosis. Compared to 10% sucrose diluent, treatment with *R. mucosa* from healthy volunteers (RmHV) significantly reduced the resultant swelling (**Figure 2A**) as well as the acanthosis and dermal thickening (**Figure 2B**). We further compared RmHV to isolates taken from patients with AD (RmAD); although these were not cultured from psoriasis patients, these isolates lack the beneficial lipid production and TNFR2 activity seen in RmHV (5). Treatment with RmAD did not produce any improvement in swelling or histologic abnormalities and interestingly induced an increase in the size and number of sebocytes (sebaceous hyperplasia) that was not seen in the diluent treated mice (**Figures 2A, B**); this suggests both a specific benefit of the health-associated isolates and a potential harm from AD-associated *R. mucosa*.

### 3.4 Psoriasis associated with carbon dioxide pollution by US zip code

**Figure 3:**
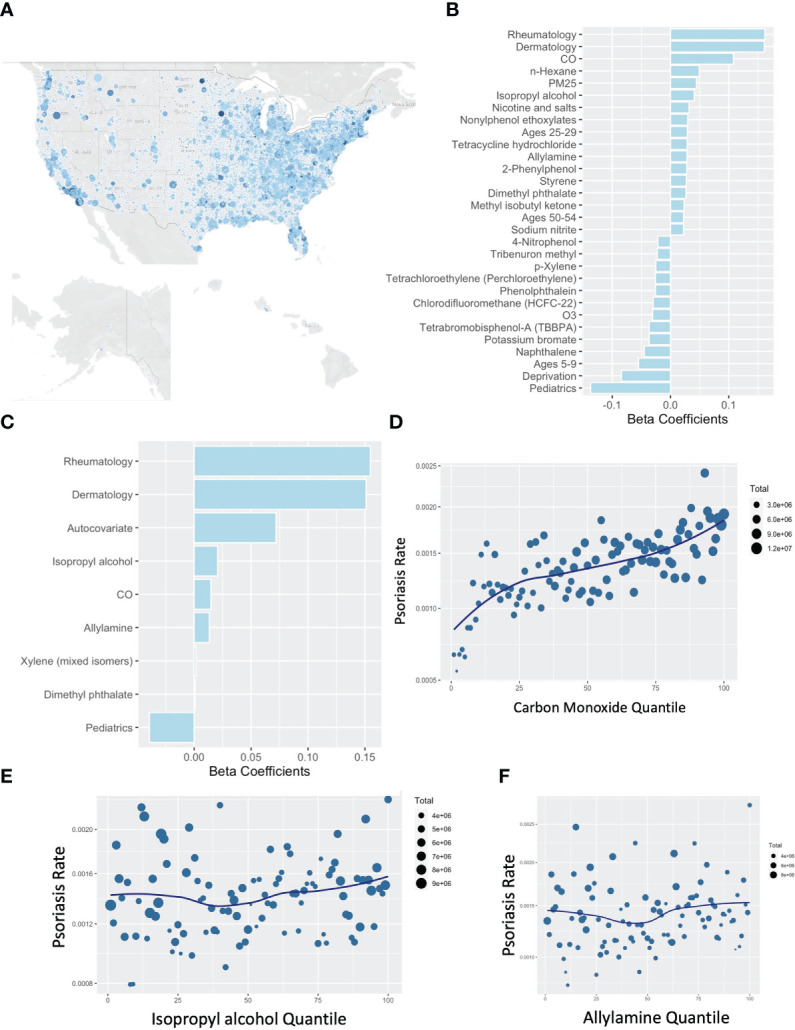
Rates of psoriasis associate with carbon monoxide by US zip code. **(A)** Map of rates of psoriasis (clinical visits billing for psoriasis divide by total billable visits) by US zip code. **(B, C)** Lasso Poisson regression models using non-spatial **(B)** or spatial **(C)** assessments pollutants associated with psoriasis. **(D–F)** Percentile plots for concentration of CO **(D)**, isopropyl alcohol **(E)**, and allylamine **(D)** against rates of psoriasis.

Similar to our previous work in AD (4), we performed an untargeted comparison between the pollution reported to the EPA under the Risk-Screening Environmental Indicators (RSEI) system. This system collates mandatory self-reporting of air pollution from facilities in the US and incorporates information about facility transfers, stack height, and wind dispersion to calculate their likely concentration across the US. In addition to the RSEI, we considered variables from the Center for Air Climate and Energy Solutions, which enriches our analysis for more common air pollutants that comprise the more familiar air quality scores. Further variables accounted for include demographic and age information from the American Community Survey (ACS) and local deprivation from the Neighborhood Atlas. Contrasting aggregate exposures from 2015-2019 against the rates of billing visits for psoriasis to medical providers in 2019 (**Figure 3A**) demonstrated a consistent association between carbon monoxide (CO) exposure and the rate of clinic visits for psoriasis as detected with a non-spatial lasso regression (**Figure 3B**), spatial lasso regression with spatially lagged y autocovariate and spatial folds (**Figure 3C**), and when visualized by pollution percentile (**Figure 3D**). Isopropyl alcohol, allylamine, and dimethyl phthalate were the other selected chemicals however quantile plots did not suggest a clear independent dose-response relationship (**Figures 3E, F**).

**Figure 4:**
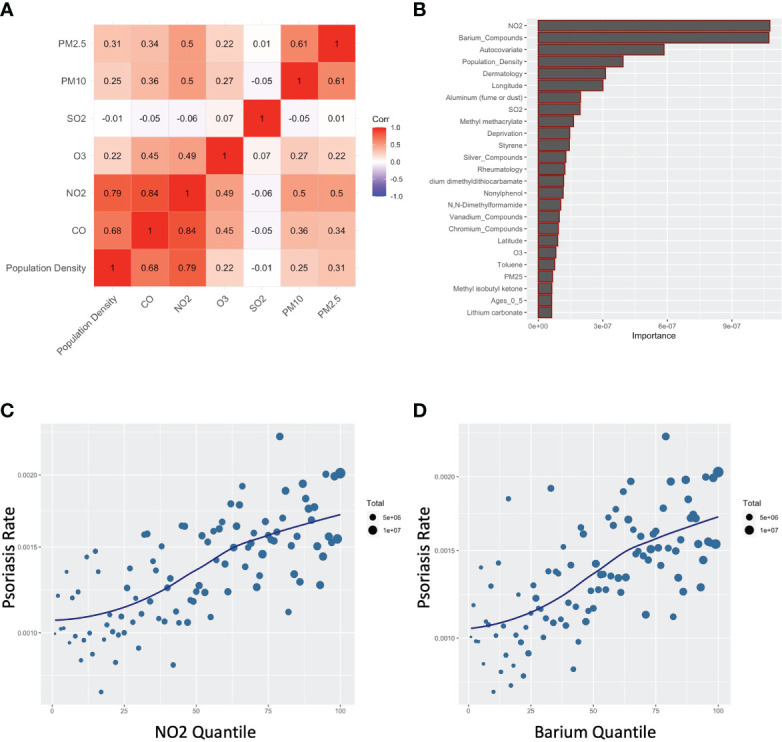
Carbon monoxide associates with other common air pollutants for psoriasis. **(A)** Correlogram for indicated pollutants. **(B)** Random forest assessment accounting for non-linearities and complex interactions **(C, D)** Percentile plots for concentration of NO2 **(C)** and barium **(D)** against rates of psoriasis.

Consistent with the literature (11, 15), there was strong co-linearity between CO and other pollutants typically associated with automobile exhaust, especially NO_2,_ but also PM2.5 and PM10 (**Figure 4A**). Furthermore, when a random forest model was used, which flexibly models nonlinear relationships and potentially complex interactions between exposures, NO_2_ was the top variable selected, along with barium (**Figure 4B**). Like CO, an independent dose-response curve was evident for both NO_2_ and barium (**Figures 4C, D**).

## 4 Discussion

TNFα has a well-established role in the pathogenesis of psoriasis. In this study, we found that *R. mucosa* had a direct effect on levels of TNFα, TACE and TNFR2 on the cell surface of primary human keratinocytes. The significant reduction of these targets on the surface of human keratinocytes can be a result of either receptor internalization or receptor shedding, either of which can occur following activation of TNFR2 (17). Further experimentation in this model will be needed to determine whether the changes in surface expression is due to shedding or internalization, but the data point to *R. mucosa* causing activation of TNFR2 signalling.

While some of the cell culture findings will require additional inquiry, direct observation of TNFR impact was seen in our clinical trial of topical *R. mucosa* for AD. However, the clinical data is limited by the small sample size and lack of placebo. Furthermore, comparison between *R. mucosa* treatment and established treatment modalities will be needed to elucidate if reductions in sTNFR2 is generally related to clinical improvement or specific to responses to *R. mucosa*. One study indicated that sTNFR levels were each associated with severity but did not assess if levels changed with clinical treatment (18).

These preclinical findings suggest that the benefit of *R. mucosa* in mouse models of psoriasis would most likely operate through either modulating TNF signalling or non-specific pathways that aide in tissue repair. Although resent evidence suggests a bidirectional association between AD and psoriasis (19), traditionally they have been viewed as having opposing immunologic findings (20) and thus finding improvement in mouse models of both diseases may be somewhat unexpected. However, the importance of both TNF and skin lipid biology have been established in the pathogenesis of psoriasis (8, 9). Therefore, even if *R. mucosa* is unlikely to specifically modulate each of the immunologic pathways that distinguish the AD and psoriasis, modulation of TNF signalling along with increased ceramide-family lipids would be expected to provide utility to any inflammatory skin disease. Therefore, our models suggest that a clinical trial using *R. mucosa* in patients with psoriasis may be worthy of consideration. In addition, our mouse models should be repeated using commensal isolates taken from patients with psoriasis as comparisons, in addition to the *Rm*AD used in our work thus far.

*R. mucosa* is not the only skin organism capable of modulating ceramide-family lipid production. For example, *Staphylococcus epidermidis* has recently been shown to enhance host production of ceramides and thus may also offer therapeutic benefit (21). While commensal influence over cutaneous TNF signalling is less well established in the literature, future work could screen isolates for impact on TNF and/or the TNF receptors. As recently well reviewed (22), microbes which as over-represented in lesional skin in psoriasis compared to controls and/or unaffected skin include *Corynebacterium* spp., *S. pyogenes*, *Neisseria* spp., and *Propionibacterium* spp. If these organisms contribute to symptom severity, then strategies which target these organisms may also provide benefit to patients.

Furthermore, the pathophysiology of psoriasis goes beyond this manuscripts TNF focus. Numerous other cytokines and chemokines are involved in the pathogenesis of psoriasis including but not limited to interleukin (IL-) 1β, IL-17, IL-23, and CXCL1 (22). *In vitro* screening for commensals which modulate any of these additional immunologic markers might identify viable probiotic options that may offer more targeted benefit and/or synergize with *R. mucosa*. However, one thing to be mindful of in researching diseases like psoriasis is the challenge presented by the potential for delayed onset of symptoms. Microbial disruption in the gut or skin present in childhood could generate disease later in life and thus there is a risk that the most valuable dysbiotic insights could fade from detection by the time of disease onset.

We used multiple models to query for psoriasis-associated pollutants. CO was ranked most important by our penalized regression models, though it may only be a marker of vehicle exhaust, urbanicity, or often-associated pollutants like NO_2_. A previous study reported that subacute exposure to pollutants often found in vehicle exhaust, including CO, NOx, PM2.5, PM10, and benzene, are associated with psoriasis flares (15). Select studies have linked CO with autoimmunity in general (23) and psoriasis in specific (15). Although average CO levels in the US have been declining during the era of rising rates of psoriasis (24), increased population growth in urban areas could still present a means for increased overall exposure to CO (11, 25). Isopropyl alcohol and allylamine were weakly associated with psoriasis, but neither have reported links to psoriasis in the literature. The permutation importance rankings from the random forest regression model, which is flexible to non-linearities and complex interactions, suggested NO_2_ (**Figure 4B**), which is highly correlated with CO (**Figure 4A**).

Thus, similar to our clinical modelling, our assessment of psoriasis-associated toxins is limited by its current speculative stage. The literature suggests possible mechanisms for CO induced inflammation through feedback loops with the host enzyme heme oxygenase 1, which is elevated in the plaques and serum of psoriasis patients and associated with metabolic syndrome (26–28). Additionally, bacterial ATP activation of the host inflammasome can be mediated by CO signalling from macrophages (29). However, given the high correlation between CO and general industrialization, and because our psoriasis database represents visits to providers aggregated by zip code rather than individual-level data, it may be plausible to conclude that the correlation with psoriasis and CO in our databases may be partly representative of the ease with which patients can transit to their providers in urbanized settings. Other researchers may also need to consider the possibility that correlations in measured CO and NO_2_ may be nonspecific associations with population density rather than a biologic mechanism of disease. Therefore, directed investigation will be needed to test if these reported findings are relevant in psoriasis. For example, patient registries containing both location and severity could be compared in a cross-sectional and/or longitudinal study against measured CO levels in the surrounding geography. If our findings can be validated, the clinical implications include the potential for improving outcomes in and/or prevention of psoriasis *via* microbial manipulation and targeted environmental mitigation. In conclusion, our findings suggest that while AD and psoriasis represent distinct pathologies, they may share a potential for future therapeutic benefit through microbial manipulation of the TNF axis and/or through environmental mitigations.

## Data availability statement

The raw data supporting the conclusions of this article will be made available by the authors, without undue reservation.

## Ethics statement

The studies involving human participants were reviewed and approved by IRB of the National Institutes of Health. Written informed consent to participate in this study was provided by the participants’ legal guardian/next of kin. The animal study was reviewed and approved by Animal Care Board for National Institutes of Health.

## Author contributions

PG performed all studies involving cell culture staining and wrote the manuscript. JZ performed the assessments for pollutants associated with psoriasis. IM performed serum and murine studies and wrote the manuscript. All authors contributed to the article and approved the submitted version.
